# Editorial: The oral microbiome and its impact on systemic health: from disease development to biomaterials development

**DOI:** 10.3389/fcimb.2025.1697069

**Published:** 2025-09-24

**Authors:** Wentao Jiang, Shensheng Gu

**Affiliations:** ^1^ Department of Endodontics, Shanghai Ninth People’s Hospital, Shanghai Jiao Tong University School of Medicine, Shanghai, China; ^2^ College of Stomatology, Shanghai Jiao Tong University, Shanghai, China; ^3^ National Center for Stomatology & National Clinical Research Center for Oral Diseases, Shanghai, China; ^4^ Shanghai Key Laboratory of Stomatology & Shanghai Research Institute of Stomatology, Shanghai, China

**Keywords:** dental biofilm, host-microbe interaction, regulatory mechanism, oral microbiota and systemic diseases, oral microbial ecology

The human body is a complex ecosystem, harboring a vast array of microorganisms that profoundly influence host health. Among these, the oral cavity stands as the second largest and most diverse microbial reservoir, playing a pivotal role in both local oral homeostasis and broader systemic well-being (Caselli et al., 2020). Emerging evidence increasingly highlights that dysbiosis of the oral microbiota is not merely confined to oral diseases but can instigate or exacerbate a spectrum of systemic conditions through intricate immune and metabolic pathways (Sedghi et al., 2021; Kunath et al., 2024). This Research Topic brings together a collection of studies that advance our understanding of these complex interplays, from specific bacterial contributions to inflammation to the impact of oral health on distant organs and neurological functions. Utilizing cutting-edge methodologies, these articles shed light on novel pathogenic mechanisms and explore promising therapeutic avenues, emphasizing the critical nexus between oral microbiota, immune dysregulation, and systemic health.

A central theme emerging from this compilation is the causal role of oral microbial dysbiosis in driving inflammatory responses. Liu et al. shed light on the specific contribution of *Escherichia coli* (*E. coli*) to oral mucosal inflammation. Their study, utilizing a novel mouse model of oral mucositis induced by tongue scratch and topical *E. coli* application, demonstrates how *E. coli* stimulation aggravates local and systemic inflammatory responses, characterized by increased expression of IL-6, IL-17, CCR6, and CCL20, and a notable imbalance in the Th17/Treg cell ratio. This research provides crucial experimental groundwork for understanding oral inflammatory diseases like oral lichen planus (OLP) from a microbiological perspective.

Extending the investigation into persistent oral infections, Li et al. employ high-performing cross-dataset machine learning to uncover robust microbiota alterations in secondary apical periodontitis (SAP). This meta-analysis not only confirms the prevalent involvement of Enterococcus faecalis in SAP but also identifies previously underestimated culprits such as Cutibacterium acnes and Delftia acidovorans. The study further elucidates functional insights by revealing the enrichment of the phosphotransferase system (PTS) and peptidoglycan biosynthesis pathways, linking these to biofilm formation and sugar metabolism in AP pathogenesis (Suriyanarayanan et al., 2018). This work underscores the power of advanced computational methods in resolving inconsistencies across studies and providing a more reproducible understanding of complex microbial communities (Gurevitch et al., 2018).

The influence of the oral microbiome extends far beyond the confines of the oral cavity, establishing critical oral-systemic axes. Ye et al. provided compelling evidence for a direct link between oral and systemic health by showing that periodontitis (PD) significantly aggravates idiopathic pulmonary fibrosis (IPF) in mice. Their findings reveal that the periodontal pathogen Porphyromonas gingivalis (Pg) is enriched in both the oral cavity and lungs, promoting the infiltration of neutrophils and Th17 cells, with Th17 cells regulating neutrophils via IL-17A. This highlights the oral-lung axis and suggests that targeting oral pathogens like Pg could be a promising strategy for ameliorating systemic conditions such as IPF. Another fascinating oral-systemic connection is explored by Deng et al., who investigate salivary signatures of oral-brain communication in sleep bruxers (SB). Through an integrated analysis of metagenomics and metabolomics, their work reveals microbial dysbiosis and altered oral metabolites in SB individuals. Key findings include a reduced level of Streptococcus mitis correlating with lower N-acetylglucosamine (GlcNAc), decreased salivary IFN-γ, and an altered IFN-γ/IL-4 ratio, alongside upregulated amino acid metabolism and neurotransmitter-associated pathways. This research proposes a neuroimmune regulatory network as a potential underlying mechanism of oral-brain communication in SB, broadening our understanding of the complex interplay between oral health and neurological function.

Given the significant impact of oral microbial dysbiosis, therapeutic strategies modulating the oral microecology are gaining traction. Li et al. provide a comprehensive review on the use of probiotics in managing radiation-induced oral mucositis (RIOM), a debilitating complication in cancer patients. They emphasize that radiotherapy induces oral microbial dysbiosis, shifting towards pathogenic gram-negative flora, which exacerbates RIOM. The review compiles evidence demonstrating that both gut-derived and oral cavity-derived probiotics can effectively reduce the incidence, duration, and severity of RIOM by modulating the microbiota and regulating host immune responses (Wang et al., 2021; Peng et al., 2024). This collective work underscores the potential of targeted microbial interventions as adjunctive therapies.

Collectively, the articles in this Research Topic reinforce the oral microbiome as a dynamic and influential entity, actively participating in a wide array of host health and disease processes. They highlight the growing importance of multidisciplinary approaches, integrating advanced methodologies like multi-omics, machine learning, and refined animal models, to unravel complex host-microbe interactions and overcome previous research challenges. These studies pave the way for a more comprehensive understanding of microbial pathogenesis and the development of precision-focused therapeutic and diagnostic strategies. Despite these significant advancements, several challenges and future directions remain. A critical area is to further establish definitive causal relationships between specific oral microbes, their metabolites, and the onset or progression of systemic diseases in human cohorts. Detailed mechanistic elucidations at the molecular and cellular levels are also needed to fully understand how microbial signals translate into host immune modulation and organ pathology. The development of personalized microbial interventions, such as probiotic strains tailored to an individual’s unique microbiome profile and host response, holds immense promise but requires extensive validation through long-term clinical trials to ensure both efficacy and safety, particularly in immunocompromised patients. Furthermore, investigating the complex synergistic and antagonistic interactions within polymicrobial communities, rather than focusing solely on single pathogens, will provide a more holistic view of disease pathogenesis. This Research Topic serves as a testament to the rapidly evolving field of oral microbiome research, providing a robust foundation for future investigations into microbial-host interactions and their profound implications for human health.
